# Estimating Pneumonia Deaths of Post-Neonatal Children in Countries of Low or No Death Certification in 2008

**DOI:** 10.1371/journal.pone.0025095

**Published:** 2011-09-22

**Authors:** Evropi Theodoratou, Jian Shayne F. Zhang, Ivana Kolcic, Andrew M. Davis, Sunil Bhopal, Harish Nair, Kit Yee Chan, Li Liu, Hope Johnson, Igor Rudan, Harry Campbell

**Affiliations:** 1 Centre for Population Health Sciences, University of Edinburgh Medical School, Edinburgh, United Kingdom; 2 School of Public Health, Medical School, University of Zagreb, Zagreb, Croatia; 3 Newcastle University and Newcastle Hospitals NHS Foundation Trust, Newcastle Upon Tyne, United Kingdom; 4 Public Health Foundation of India, New Delhi, India; 5 Nossal Institute for Global Health, University of Melbourne, Melbourne, Australia; 6 School of Public Health, Peking University, Beijing, China; 7 Department of International Health, Johns Hopkins University Bloomberg School of Public Health, Baltimore, Maryland, United States of America; 8 Croatian Centre for Global Health, University of Split Medical School, Split, Croatia; Institut Pasteur, France

## Abstract

**Background:**

Pneumonia is the leading cause of child deaths globally. The aims of this study were to: a) estimate the number and global distribution of pneumonia deaths for children 1–59 months for 2008 for countries with low (<85%) or no coverage of death certification using single-cause regression models and b) compare these country estimates with recently published ones based on multi-cause regression models.

**Methods and Findings:**

For 35 low child-mortality countries with <85% coverage of death certification, a regression model based on vital registration data of low child-mortality and >85% coverage of death certification countries was used. For 87 high child-mortality countries pneumonia death estimates were obtained by applying a regression model developed from published and unpublished verbal autopsy data from high child-mortality settings. The total number of 1–59 months pneumonia deaths for the year 2008 for these 122 countries was estimated to be 1.18 M (95% CI 0.77 M–1.80 M), which represented 23.27% (95% CI 17.15%–32.75%) of all 1–59 month child deaths. The country level estimation correlation coefficient between these two methods was 0.40.

**Interpretation:**

Although the overall number of post-neonatal pneumonia deaths was similar irrespective to the method of estimation used, the country estimate correlation coefficient was low, and therefore country-specific estimates should be interpreted with caution. Pneumonia remains the leading cause of child deaths and is greatest in regions of poverty and high child-mortality. Despite the concerns about gender inequity linked with childhood mortality we could not estimate sex-specific pneumonia mortality rates due to the inadequate data. Life-saving interventions effective in preventing and treating pneumonia mortality exist but few children in high pneumonia disease burden regions are able to access them. To achieve the United Nations Millennium Development Goal 4 target to reduce child deaths by two-thirds in year 2015 will require the scale-up of access to these effective pneumonia interventions.

## Introduction

Pneumonia is the leading cause of death in children under the age of 5 years, with more than 98% of pneumonia deaths occurring in developing countries [1]. Among children who survive, pneumonia is a major cause of illness globally with an estimated 156 million new episodes occurring each year, 8% of which are severe and require hospitalization [2]. In 2009, a Global Action Plan for Prevention and Control of Pneumonia (GAPP) was launched by the World Health Organization (WHO), UNICEF and partners, with the aim of increasing awareness of pneumonia as a major cause of death and promoting the use of interventions with proven effectiveness [3]. In 2010 the World Health Assembly recognised the importance of pneumonia as a global health problem and passed the resolution “Accelerating progress towards achievement of Millennium Development Goal 4 (MDG4) to reduce mortality: prevention and control of pneumonia”. Quantifying the burden of disease due to pneumonia is essential to monitor progress towards MDG4.

Since many high under-five mortality rate (U5MR) countries lack complete death registration, several attempts have been made to estimate the burden of child pneumonia mortality using single- or multi-cause statistical models. In the single-cause model, the proportion of pneumonia deaths is estimated using a log-linear regression that can include several covariates. Previously, a single-cause model published in 2002 estimated that child deaths from pneumonia accounted for approximately 19% of all deaths for the year 2000 [4]. In the multi-cause model one cause of death is selected as the base cause and the ratio of the proportion of each of the other causes of death relative to the proportion of the ‘base’ cause is estimated using an ordinary least squares regression [5]. A multi-cause model estimate for countries without adequate vital registration systems published in 2003 estimated that child pneumonia deaths accounted for 23% of all child deaths in sub-Saharan Africa and South Asia for the year 2000 [6]. More recently, multi-cause model based estimates were published and suggested that neonatal and post-neonatal pneumonia deaths accounted for 18% of all child deaths for the year 2008 [1].

In this paper, we provide year 2008 pneumonia mortality estimates for children 1–59 months for countries with low (<85%) or no coverage of death certification (n = 122 countries) using a single-cause model similar to that used to provide year 2000 estimates previously [4]. We also compare the single-cause pneumonia mortality estimates with those recently published using a multi-cause model [1].

## Methods

### Pneumonia mortality post-neonatal estimates

The number of post-neonatal pneumonia deaths for 122 countries was estimated using two different methods according to the U5MR and gross national income per capita at purchasing power parity (GNI PPP) of the countries. A detailed description for each step is presented in **Table 1** and, **Supplementary panel S1**.

**Table 1:** Methods for estimating pneumonia child mortality in all countries (U5MR: under five mortality rate, GNI PPP: gross national income per capita at purchasing power parity, VR: vital registration, HIV ANC: index score for HIV prevalence based on the antenatal care surveillance; China U5MR in 2008 was 20.5/1000lb, however it was included in the Verbal autopsy model given that this country's profile is closer to the one of the high mortality countries and its GNI PPP was <$7510 in 2008).

	Vital registration model (35 countries)	Verbal autopsy model(87 countries)
**Countries**	a. U5MR <26/1000lb or GNI PPP >$7510b. VR coverage <85%	U5MR ≥26/1000lb
**Sources**	VR data from low mortality - high coverage countries	a. PubMed search using the following keywords:*Pneumonia, mortality*b. Published from 1980-01-01 to 2008-11-01
**Inclusion criteria**	n/a	a. Estimated pneumonia proportionate mortality in children 0–4yrs in developing countries and reported overall U5MR at the study siteb. Were community-based and longitudinal OR national based data OR control arms of intervention trialsc. Had a duration of at least 12 months (prospective or retrospective studies) OR included subjects of exactly the same age range (birth cohorts)d. Used verbal autopsies to assign the cause of deathe. Estimated mortality from at least two unique causesf. Had reported more than 50 deaths and had no more than 1/3 of deaths attributed to undetermined causes
**Model**	Single cause regression model including the following covariates: U5MR, GNI PPP and WHO area	Single cause regression model including the following covariates:U5MR, HIV ANC, malaria prevalence, two variables for the lower and upper age bounds

### Vital registration model (VRM) for countries of low mortality (U5MR) and low VR coverage

For 35 countries with U5MR<26 per 1000 live births or of GNI PPP >$7,510 (international dollars) but inadequate death registration (<85%), estimates of post-neonatal pneumonia deaths and 95% confidence intervals (95% CI) were produced using a single-cause regression model that used death registration data from 64 low mortality countries with high quality VR data (extracted from the WHO Mortality Database using the International Classification of Diseases 9 and 10 versions). This model included covariates for U5MR, GNI PPP per capita and a regional indicator variable for WHO classification regions (**Supplementary panel S1**).

### Verbal autopsy models (VAM) for countries of high mortality (U5MR)

For 87 countries with U5MR≥26 per 1000 live births pneumonia deaths and 95% CI were estimated based on single cause regression models developed from verbal autopsy studies (VAM). China's U5MR in 2008 was 20.5/1000 LB. However China was included in this category given that this country's profile is closer to the one of the high mortality countries and its GNI PPP was <$7510 in 2008.

#### Search strategy

The search strategy that was used to identify verbal autopsy studies and the inclusion criteria of the selected ones is presented in **Table 1**. Included studies were community-based, longitudinal multi-cause studies (studies reporting at least two different causes of death) or control arms of clinical trials of child mortality. Those studies were obtained through a literature search of Pubmed for the period between 1980 and 2009, using the keywords “pneumonia” and” mortality” and restricting the search to children. The search returned 2234 papers for further evaluation. We first excluded duplicate studies and studies that did not estimate mortality in children 0–4yrs in developing countries and did not report overall U5MR at the study site. We then excluded any studies that were not community-based and were not longitudinal or national based data or control arms of intervention trials. In the next step we excluded all studies that did not have duration of at least 12 months (prospective or retrospective studies) or did not include subjects of exactly the same age range (birth cohorts). We then excluded all studies that did not use verbal autopsies to assign the cause of death. In the next step we excluded any single-cause studies, as single cause studies tend to over-estimate the cause of death they are examining. Finally, we excluded studies with fewer than 50 deaths and studies that had more than 1/3 of deaths attributed to undetermined causes, adhering to standard Child Health Epidemiology Reference Group (CHERG) methods [7], [8]. Eventually, we included 58 studies (81 data points) published between 1986 and 2008 for statistical modeling. The geographic location of the 81 data points is shown in **Supplementary figure S1**. In brief, 37 studies were conducted in South East Asian region, 25 were conducted within the African region, 10 were conducted in the Americas region, seven were conducted in the East Mediterranean region and two were conducted in the West Pacific region.

Some studies reported also malnutrition as a cause of death. These malnutrition deaths were reallocated to other related (infectious) causes of death that were reported in the study (e.g. pneumonia, diarrhea, malaria) [6]. In addition, in some studies multi-cause deaths were reported (for example one child had as cause of death diarrhea and pneumonia). These multi-cause deaths were reallocated to each related cause according to the proportion of the single-cause deaths in the study (for example: one study reported 80 pneumonia deaths, 60 diarrhea deaths and 20 pneumonia-diarrhea deaths. Of the 20 multi-cause deaths 11 were re-distributed to the pneumonia deaths and 9 to the diarrhea deaths based on the proportion of the single cause pneumonia and diarrhea deaths (80/60 = 1.3)).

#### Verbal autopsy models

The number of pneumonia deaths for children aged 1–59 months in the 85 high mortality countries was estimated using two single cause regression models. For the first one we used studies that were conducted in sub-Sahara African settings (VAM_1a_) and for the second we used studies that were conducted in settings of developing countries not in the sub-Sahara region (VAM_1b_). The covariates for both models were U5MR, an index score for malaria prevalence (described in detail in [5]), an index score for HIV prevalence based on the antenatal care (ANC) surveillance and two variables for the lower and upper age bounds. Studies were given a weight proportional to the square root of the number of deaths on which they had data. The models were then populated with year 2008 country-level covariate data in order to estimate the proportion of pneumonia deaths for the high mortality countries. The HIV ANC score of the studies that were included to develop the models ranged from 0 to 7. However eight sub-Saharan countries had an HIV ANC score above 7. For these eight countries we used a model that did not include the HIV covariate and when populating the model we used their HIV-free envelopes of all deaths (**Supplementary panel S1**).

### Regional and national estimates

Country-level post-neonatal pneumonia estimates (derived either from the VR or VA models) were adjusted post-hoc for the use of *Haemophilus influenzae* type b (*Hi*b) vaccine, in order to account for the impact of *Hi*b vaccination scale-up in 2008. In particular, for each country we extracted the number of possible prevented number of post-neonatal *Hi*b pneumonia deaths, which were estimated by multiplying the vaccine immunization coverage for three doses of Hib (Hib3; extracted from UNICEF, [9]) by the vaccine efficacy of Hib3 against chest X-ray confirmed pneumonia [10]. Regional estimates of post-neonatal pneumonia deaths were obtained by adding together the country-level data for the six WHO regions.

### Sensitivity analysis

We conducted several sensitivity analyses to check how model-dependent the estimates were. In particular for the 87 high mortality countries we applied (i) two VA models (one for the sub-Sahara high mortality countries and one for the remaining high mortality countries) adjusted only for U5MR and the two age-related variables (VAM_2a_ and VAM_2b_); (ii) one global model (for all 85 high mortality countries) adjusted for U5MR, an index score for malaria prevalence, an index score for HIV prevalence based on ANC surveillance and the two age-related variables (VAM_3_); and (iii) one global model (for all 85 high mortality countries) adjusted only for U5MR and the two age-related variables (VAM_4_). In addition, we conducted a total, regional and national comparison between the current single-cause based estimates and the multi-cause based estimates of the year 2008 [1].

## Results

### Vital registration model (VRM) for countries of low mortality

The parameter estimates of the VR model are presented in **Table 2**. For the year 2008, there were 35 countries of low mortality but with low VR data coverage (**Supplementary table S1**) and their post-neonatal child pneumonia deaths were estimated using the VR model. The U5MR for the 35 VRM countries ranged from 3.6/1000 LB in Andorra to 67.1/1000 LB in South Africa.

**Table 2:** Parameter estimates for the vital registration and verbal autopsy models (lnU5MR: Natural logarithm of the under 5 mortality rate; GNI: Gross National Income; WHO: World Health Organisation).

Models	Predictors	ParameterEstimate	R^2^
**Vital registration model**	lnU5MR	0.17	0.52
	GNI	−0.00	
	WHO region	−0.18	
***Verbal autopsy models***			
Model for Sub-Sahara African countries	lnU5MR	0.24	0.41
	Malaria	−0.40	
	HIV	−0.04	
Model for non Sub-Sahara African high mortality countries	lnU5MR	0.32	0.32
	Malaria	0.39	
	HIV	−0.09	

### Verbal autopsy models (VAM) for countries of high mortality

The parameter estimates of the two VA models are presented in **Table 2**. For the year 2008, there were 87 high mortality countries (**Supplementary table S1**) and their post-neonatal pneumonia deaths were estimated using the VA models. The U5MR for the 85 VAM countries ranged from 20.5/1000 LB in China to 257/1000 LB in Afghanistan. The VAM_1a_ was populated for 44 high mortality sub-Sahara African countries and the VAM_1b_ was populated for the 43 remaining high mortality developing countries.

### Total, regional and national estimates

**Figure 1:**
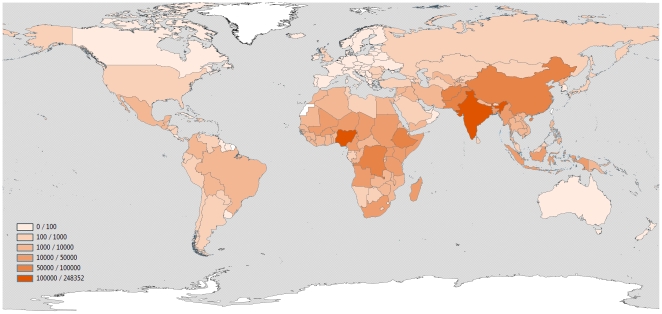
National estimates of number of pneumonia deaths for children 1–59 months (data on 71 low mortality and high vital registration coverage are extracted from the WHO Mortality Database using the International Classification of Diseases 9 and 10 versions).

**Figure 2:**
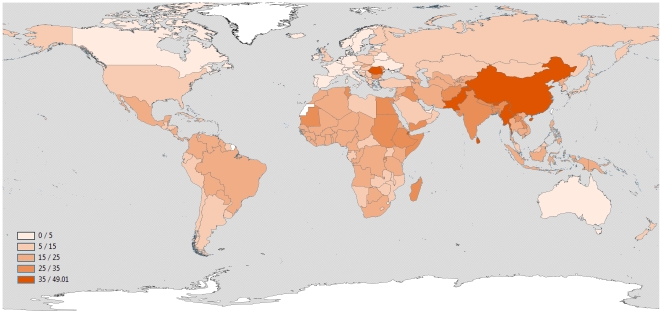
National estimates of % pneumonia deaths for children 1–59 months (data on 71 low mortality and high vital registration coverage are extracted from the WHO Mortality Database using the International Classification of Diseases 9 and 10 versions).

Total post-neonatal pneumonia deaths for the year 2008 were estimated to be 1.18 M (95% CI: 0.77 M, 1.80 M), accounting for 23.27% (95% CI: 17.15%, 32.75%) of all post-neonatal deaths (**Table 3**). WHO regional post-neonatal mortality estimates are presented in **Table 3**. The countries with the highest absolute and relative post-neonatal pneumonia mortality are presented in **Table 4** and national estimates of absolute and relative pneumonia mortality for post-neonatal children are presented in **Supplementary table S1** and **Figures 1** and **2**.

**Table 3:** Post-neonatal pneumonia number of deaths and mortality rates for the WHO regions (PN: Pneumonia; 95% CI: 95% Confidence Interval; WHO: World Health Organisation).

Regions	# of 1–59m PN deaths (95% CI)	# of 1–59m PN deaths over 1–59m total deaths (95% CI)
**122 countries**	1181037(773857, 1803686)	23.27%(17.15%, 32.75%)
**WHO region**		
Countries from Africa WHO region (n = 44 of 46)	569940(358622, 896048)	19.22%(12.09%, 30.22%)
Countries from America WHO region (n = 15 of 35)	9149(5218, 15706)	16.59%(9.46%, 28.47%)
Countries from East Mediterranean WHO region (n = 19 of 21)	207061(141241, 306405)	30.69%(20.93%, 45.41%)
Countries from Europe WHO region (n = 15 of 53)	6983(4470, 10945)	16.01%(10.25%, 25.09%)
Countries from South East Asia WHO region (n = 11 of 11)	336329(234801, 484244)	31.45%(21.96%, 45.29%)
Countries from West Pacific WHO region (n = 18 of 27)	51575(29504, 90337)	20.79%(11.89%, 36.42%)

**Table 4:** Countries with the highest absolute and relative post-neonatal pneumonia mortality ordered by rank.

Rank	# of 1–59m PN deaths	% of 1–59m PN deaths over 1–59m total deaths
1	India	Cape Verde
2	Nigeria	Pakistan
3	Dem. Rep. of the Congo	Bhutan
4	Pakistan	Timor-Leste
5	Afghanistan	Lesotho
6	Ethiopia	Nauru
7	China	Myanmar
8	Kenya	Djibouti
9	Sudan	Sri Lanka
10	Angola	Bangladesh
11	Bangladesh	Somalia
12	Indonesia	Mauritania
13	Uganda	India
14	United Republic of Tanzania	Nepal
15	Myanmar	Sudan
16	Niger	Yemen
17	Somalia	Ethiopia
18	Burkina Faso	Comoros
19	Chad	Egypt
20	Mali	Sao Tome and Principe

The results of the sensitivity analysis of the 87 high mortality countries are presented in **Table 5**. Post neonatal pneumonia death estimates were consistent with 3 out of the 4 approaches predicting an estimate of 1.17 M to 1.18 M post-neonatal pneumonia deaths. The total, regional and national comparison between the current single-cause based estimates and the multi-cause based estimates of the year 2008 [1] are presented in **Supplementary table S2**. Both models estimated that child pneumonia deaths accounted for 23% of all cause post-neonatal child deaths of the 122 countries. In terms of regional differences, the estimates from both models were relatively consistent for countries from the Africa, East Mediterranean and South East Asia WHO regions but not for countries from the America, Europe and West Pacific WHO regions (with estimates varying by 1.2%, 4.46%, 5.57%, 9.38%, 14.08% and 13.76% respectively; **Supplementary table S2**). Finally, the correlation coefficient for the agreement between the national estimates was 0.40 (**Supplementary Figure S2**).

**Table 5:** Sensitivity analysis for the global post-neonatal pneumonia estimates based on the application of alternative verbal autopsy models (VAM) for the high mortality countries (Number of pneumonia deaths for low mortality countries and for India and China are estimated in the same way among all four different approaches; VAM1a & VAM1b are the models presented in the main analysis; PN: Pneumonia; lnU5MR: Natural logarithm of the under 5 mortality rate; HIV ANC: index score for HIV prevalence based on the antenatal care surveillance).

Models	Model description	Global 1–59 m PN deaths(% of all 1–59 m deaths)
VAM1a & VAM1b	2 VA models (one for the sub-Sahara high mortality countries and one for the remaining high mortality countries) adjusted for lnU5MR, an index score for malaria prevalence, the HIV ANC score and two age-related dummy variables (model presented in the main analysis)	***1.18M (23.33%)***
VAM_2a_ & VAM_2b_	2 VA models (one for the sub-Sahara high mortality countries and one for the remaining high mortality countries) adjusted for lnU5MR and two age-related dummy variables	1.17 M (23.12%)
VAM_3_	1 global model (for all 85 high mortality countries) adjusted for lnU5MR, malaria prevalence, HIV ANC and the two age-related dummy variables (VAM_3_)	1.04 M (20.55%)
VAM_4_	1 global model (for all 85 high mortality countries) adjusted only for lnU5MR and the two age-related dummy variables	1.18 M (23.33%)

## Discussion

### Estimates

We estimated that there were 1.18 M (95% CI 0.77 M, 1.80 M) post-neonatal pneumonia deaths in the year 2008, representing 23.27% (95% CI: 17.15%, 32.75%) of all post-neonatal deaths in 122 countries of low or no death certification. The WHO region with the highest number of pneumonia deaths was the African region (with 569,940 post-neonatal pneumonia deaths), whereas the regions with the highest percentage of post-neonatal pneumonia deaths were the East Mediterranean and South East Asian regions (with 30.69% and 31.45% of all post-neonatal deaths respectively). The countries with the highest number of pneumonia deaths were India, Nigeria, Democratic Republic of the Congo, Pakistan, Afghanistan and Ethiopia and these accounted for more than 55% of total pneumonia deaths.

We elected to account for malaria and HIV infection in our models. The assumption is that in areas where malaria is highly prevalent (mainly in countries of the Sub-Saharan Africa), malaria's attributable mortality will be high and thus needs to be taken into account. In addition, low birth weight because of maternal malaria infection may make the child more susceptible to poor prognosis from childhood infections. Thus malaria needs to be taken into account as an alternative cause of child death and as an indirect contributor through low birth weight. With regard to HIV, we included HIV prevalence in our models for similar reasons (i.e. HIV's attributable mortality in countries of high prevalence, increased susceptibility of HIV+ children to and increased mortality from infections including pneumonia). However, there is limited information about the effect of HIV infection on childhood pneumonia related morbidity and mortality and further research needs to be conducted.

It is notable that despite general concerns about gender inequity linked with childhood mortality we could not estimate sex-specific pneumonia proportionate mortality due to inadequate data. In particular, we could only identify four studies that reported child mortality in girls and boys separately. Gender inequity could occur with parents being more likely to seek care and receive treatment for sick sons than daughters [11]. We recommend that future studies report (pneumonia) mortality rates separately for boys and girls so that investigation of these issues can be conducted.

### Temporal trends in pneumonia mortality

When comparing the number of estimated child pneumonia deaths between the years 2000–2003 [12] and 2008, we observed a 27% reduction in pneumonia mortality. This reduction may be explained by a general decrease in the overall child mortality in developing countries. In particular, in the years 2000–2003 the average annual total number of child deaths under five years old was 10.4 M, whereas it was 8.8 M deaths in 2008 [13], corresponding to a decline of 15%. Given the reduction in U5MR over this period, the fall in the proportion of child deaths due to pneumonia can be explained in part by the clear observed association between overall U5MR and the proportion of under five deaths due to pneumonia. This relationship is likely to be multifactorial and due in part to general socio-economic development (and associated changes in factors such as fertility and maternal education and empowerment) and in part due to development of general health services infrastructure and specific health programmes. In recent years the latter has included the introduction of effective new vaccines against bacterial pneumonia such as *Hi*b vaccine and pneumococcal conjugate vaccines (PCVs), however these vaccines have been implemented mainly in countries of lower mortality and therefore these interventions probably had a limited impact on global pneumonia mortality estimates [14]. In particular, for the year 2008, none of the 87 high mortality countries (including India and China) had introduced the PCV in their immunisation programmes, and only 41 of them had introduced the HibCV, with the average coverage being 75% (ranging from 10% to 99%). In addition, none of the six countries accounting for 55% of all child pneumonia deaths and only 8 of the 20 high pneumonia mortality countries (**Table 4**) had introduced a HibCV vaccination programme (in 2008).

In addition to the overall mortality fall and the socio-economic developments, the differences that we observe between the years 2000–2003 and 2008 might also be due (at least partly) to differences in the estimation methods used and therefore should be interpreted with caution. In particular, the methods that were used for the production of the current pneumonia mortality estimates are different in terms of a) the way the under five mortality envelope is estimated, b) splitting the neonatal and post-neonatal age periods and c) the way that the proportion of pneumonia mortality is estimated (see the section improvement from previous single cause based estimate for more details).

### Differences between multi and single-cause based estimates of post-neonatal pneumonia mortality

We compared the national, regional and total single-cause and multi-cause based estimates for the year 2008 [1]. The correlation between the two sets of national post-neonatal estimates was 0.40. In terms of regional differences, the estimates from both models were relatively consistent for the Africa, East Mediterranean and South East Asia regions but not for the America, Europe and West Pacific regions. Both models estimated that post-neonatal pneumonia deaths accounted for 23% of all cause deaths in these 122 countries. The global single-cause and multi-cause based estimates for the proportion of child deaths due to pneumonia for the year 2008 differed by only 0.35% in absolute and 1.5% in relative terms. This illustrates a general property of these mortality models that they yield the most stable and reliable estimates at the total and regional level but generally should be used for national planning or monitoring purposes only with great caution.

### Improvement from previous single cause based estimate (high mortality countries)

The previous single-cause based pneumonia mortality estimate was based on a model developed by Williams et al in 2002 [4]. Although the authors tried to summarise the evidence in the best possible way there were a few problems that we tried to minimise in this estimate. In particular, the VA studies that were used to develop the model (i) were not selected after a truly systematic review of the literature, (ii) there was a disproportionate number of studies from South Africa, (iii) the intervention arms of controlled trials were included, (iv) single-cause studies were not excluded and (v) it was not clear whether the selected studies included neonatal deaths and/or neonatal pneumonia deaths. In the current estimate, we conducted a literature review to find all relevant studies. We also looked through all the previous reviews to identify any studies that were not picked up by our search. We included only the control arms of controlled trials and any single-cause pneumonia studies were excluded to avoid introduction of bias due to relative over-reporting of pneumonia deaths. Finally, each study was screened thoroughly and it was coded according to whether or not it included neonatal deaths.

In relation to the development of the model, (i) the only covariate that was used in the previous estimate was U5MR and (ii) the same model was used to predict the number of pneumonia deaths for all high mortality countries. In the current estimate we used two additional covariates (one for HIV and one for malaria prevalence). In addition we ran several other models including other additional covariates such as percentage of urban population and an insecticide treated bed-net (ITN) score but these covariates were not included in the final model since their inclusion did not increase the percentage of the variance explained. We also developed separate models for the sub-Saharan African countries and for the remaining high mortality countries since the absolute proportions of the main causes of child death in Africa is different to other high mortality settings, mainly due to the significant proportion of child deaths due to malaria in Africa but not in other global regions.

### Conclusions

We estimated that 1.18 M post-neonatal children died due to pneumonia in the year 2008 in 122 countries of low or no vital registration coverage. The total and regional mortality estimates proved to be consistent across two (single and multi-cause) modelling approaches. The accuracy and consistency of these models may be improved further by adding covariates (e.g. risk factors such as breastfeeding prevalence, exposure to indoor air pollution and access to antibiotic treatment) that are associated with pneumonia mortality, as these data become available. In addition future models should take into account factors that will affect future pneumonia and overall child mortality such as the coverage of pneumococcal and Hib conjugate vaccine coverage, indicators of paediatric HIV infection and antiretroviral treatment, antibiotic resistance and access to health care. There is currently very poor reporting of gender-specific mortality rates and, given recent concerns over gender health inequities, we recommend that future studies give greater attention to reporting these data.

The use of global child mortality modelling approaches, such as those described here, which are based on the best possible locally appropriate data, can contribute to monitoring of the achievement of global child mortality targets. Despite the reduction in child pneumonia deaths between the years 2002 and 2008, pneumonia still remains one of the main causes of child deaths. The 63^rd^ World Health Assembly resolution on the control of child pneumonia represented an important contribution. Achievement of MDG4 resulting in a 67% reduction of pneumonia deaths by 2015, will correspond to the avoidance of more than a cumulative 5 million child deaths from pneumonia over the period between 2010 and 2015. In order to achieve MDG4 it will be important to continue to reduce the number of child pneumonia deaths, which will require scale-up of access to effective pneumonia interventions and development of interventions to reduce neonatal pneumonia mortality.

## Supporting Information

Panel S1 Detailed description of the applied methods for estimating the number and proportion child pneumonia deaths.
(DOC)

Table S1 National post-neonatal pneumonia number of deaths and mortality rates (VAM, verbal autopsy model; VRM, vital registration model; * Countries that had and HIV ANC score >7. The VAM used did not include and HIV covariate and when populating the model their HIV-free envelopes were used).
(DOC)

Table S2 Differences in the national and regional pneumonia mortality single and multi-cause estimates (Emr: Eastern Mediterranean Region; Eur: Europe Region, Afr: Africa Region; Amr: Americas Region; Sear: South East Asia Region; Wpr: Western Pacific Region; PN: Pneumonia).
(DOC)

Figure S1 Distribution of the 81 data points (58 verbal autopsy studies) that were used for the development of the single cause models.
(TIF)

Figure S2 Comparison of post-neonatal pneumonia estimates for 122 countries between the single-cause and multi-cause model estimates (as published in Black et al, 2010).
(TIF)
