# Supplementary material for: Estimating Pneumonia Deaths of Post-Neonatal Children in Countries of Low or No Death Certification in 2008
Source: PLoS One. 2011 Sep 22;6(9):e25095. doi: 10.1371/journal.pone.0025095 (PMC3178589; doi:10.1371/journal.pone.0025095)
Supplement: Panel S1 — Detailed description of the applied methods for estimating the number and proportion child pneumonia deaths. (DOC) [file pone.0025095.s001.doc]

Supplementary panel S1: Detailed description of the applied methods for estimating the number and proportion child pneumonia deaths

| **Steps** | **Task** | **Description** |
| --- | --- | --- |
| Step 1 | Post-neonatal number of pneumonia deaths for low under five mortality (U5MR<26 per 1000 live births (LB)) and high vital registration (VR) countries (≥85%) were downloaded from World Health Organisation (WHO) (n=71) | VR data were directly extracted from the WHO mortality databases using the international classification of diseases versions 9 and 10. |
| Step 2 | Estimation of post-neonatal number of pneumonia deaths for low mortality (U5MR<26 per 1000LB) or gross national income per capita at purchasing power parity (GNI PPP) >$7510 but low VR data coverage countries (<85%) (n=35) | A single-cause log linear model was developed based on death registration data from 64 low mortality countries of high quality VR data. This model included covariates for U5MR, GNI PPP per capita and regional indicator variable for WHO classification regions: 1 for Africa, East Mediterranean and South East Asia regions; 2 for America and West Pacific regions; and 3 for Europe region. This model was then populated with national specific data for the 35 countries of this category in order to estimate their post-neonatal proportions of pneumonia deaths. The proportions of pneumonia deaths were then applied to the WHO derived total number of deaths country envelopes to estimate the number of post-neonatal pneumonia deaths for these 35 countries. |
| Step 3 | Estimation of post-neonatal number of pneumonia deaths in high mortality countries (U5MR≥26 per 1000LB) (n=87) | Two single-cause log linear regression models were developed based on 81 data points of studies published between 1986 and 2008 in order to estimate post-neonatal estimates for 87 high mortality countries. The first model used studies conducted in high child-mortality settings in the sub-Saharan region and the second using studies conducted in high child-mortality settings out with the sub-Saharan region. The covariates for both models were U5MR, an index score for malaria prevalence (described in detail in Johnson et al, 2010), an index score for HIV prevalence based on the antenatal care (ANC) surveillance and two variables for the lower and upper age bounds. For eight sub-Saharan (Swaziland, Lesotho, Zambia, Namibia, Mozambique, Zimbabwe, Malawi, Central African Republic) with an HIV ANC score out with the range of the HIV ANC score of the data points used to develop the model, we used a model that did not include the covariate of HIV prevalence. Studies were given a weight proportional to the square root of the number of deaths on which they had data. The models were then populated with year 2008 country-level covariate data in order to estimate the proportion of post-neonatal pneumonia deaths for the high mortality countries. The proportion of pneumonia deaths was then applied to WHO derived total number of deaths country envelopes to estimate the number of post-neonatal pneumonia deaths for the high mortality countries. To estimate the number of the post-neonatal pneumonia deaths for the eight sub-Saharan countries, for which we used the single-cause log linear model without an HIV prevalence covariate, we used the HIV-free WHO-derived envelope. |
