# Supplementary material for: Estimating Pneumonia Deaths of Post-Neonatal Children in Countries of Low or No Death Certification in 2008
Source: PLoS One. 2011 Sep 22;6(9):e25095. doi: 10.1371/journal.pone.0025095 (PMC3178589; doi:10.1371/journal.pone.0025095)
Supplement: Table S1 — National post-neonatal pneumonia number of deaths and mortality rates (VAM, verbal autopsy model; VRM, vital registration model; * Countries that had and HIV ANC score >7. The VAM used did not include and HIV covariate and when populating the model their HIV-free envelopes were used). (DOC) [file pone.0025095.s002.doc]

Supplementary table S1 National post-neonatal pneumonia number of deaths and mortality rates (VAM, verbal autopsy model; VRM, vital registration model; Emr: Eastern Mediterranean Region; Eur: Europe Region, Afr: Africa Region; Amr: Americas Region; Sear: South East Asia Region; Wpr: Western Pacific Region; PN: Pneumonia; * Countries that had and HIV ANC score > 7. The VAM used did not include and HIV covariate and when populating the model their HIV-free envelopes were used)

| **Country** | **WHO**  **region** | **Method** | **# of 1-59m PN deaths**  **(95% CI)** | **% of 1-59m PN deaths over 1-59m total deaths**  **(95% CI)** |
| --- | --- | --- | --- | --- |
| Afghanistan | Emr | VAM | 65898 (46445, 93497) | 27 (19, 38) |
| Albania | Eur | VRM | 56 (38, 83) | 12 (8, 17) |
| Algeria | Afr | VAM | 2715 (1652, 4290) | 21 (13, 33) |
| Andorra | Eur | VRM | 0 (0, 0) | 0 (0, 0) |
| Angola | Afr | VAM | 26409 (14941, 44593) | 20 (12, 35) |
| Antigua and Barbuda | Amr | VRM | 1 (0, 1) | 9 (6, 12) |
| Armenia | Eur | VRM | 56 (33, 96) | 12 (7, 21) |
| Azerbaijan | Eur | VAM | 652 (428, 993) | 21 (14, 32) |
| Bangladesh | Sear | VAM | 22448 (16199, 31107) | 33 (23, 45) |
| Benin | Afr | VAM | 4562 (2753, 7339) | 16 (10, 26) |
| Bhutan | Sear | VAM | 262 (103, 670) | 39 (15, 99) |
| Bolivia | Amr | VAM | 1438 (962, 2101) | 19 (13, 27) |
| Bosnia and Herzegovina | Eur | VRM | 22 (13, 34) | 9 (6, 15) |
| Botswana | Afr | VRM | 141 (79, 250) | 20 (11, 35) |
| Burkina Faso | Afr | VAM | 13944 (9141, 20761) | 15 (10, 23) |
| Burundi | Afr | VAM | 6943 (2997, 14447) | 21 (9, 44) |
| Cambodia | Wpr | VAM | 5005 (3011, 8319) | 24 (14, 39) |
| Cameroon | Afr | VAM | 11172 (7532, 16571) | 17 (11, 25) |
| Cape Verde | Afr | VAM | 73 (34, 149) | 49 (23, 100) |
| Central African Republic* | Afr | VAM | 3202 (2256, 4564) | 17 (12, 24) |
| Chad | Afr | VAM | 13351 (8712, 20394) | 17 (11, 27) |
| China | Wpr | VAM | 32220 (18065, 57464) | 20 (11, 36) |
| Comoros | Afr | VAM | 382 (247, 591) | 28 (18, 43) |
| Congo | Afr | VAM | 1945 (1324, 2859) | 17 (12, 25) |
| Côte d'Ivoire | Afr | VAM | 8469 (5566, 12886) | 17 (11, 26) |
| Cyprus | Eur | VRM | 2 (1, 6) | 11 (3, 29) |
| Dem. Peoples's Rep. of Korea | Sear | VAM | 2203 (1590, 3052) | 25 (18, 34) |
| Dem. Rep. of the Congo | Afr | VAM | 79222 (53666, 116948) | 20 (14, 30) |
| Djibouti | Emr | VAM | 499 (194, 1126) | 36 (14, 82) |
| Dominican Republic | Amr | VRM | 458 (245, 813) | 14 (8, 25) |
| Ecuador | Amr | VRM | 556 (335, 895) | 15 (9, 23) |
| Egypt | Emr | VRM | 4864 (2614, 9050) | 28 (15, 51) |
| El Salvador | Amr | VRM | 193 (120, 299) | 15 (9, 23) |
| Equatorial Guinea | Afr | VAM | 474 (332, 675) | 19 (13, 27) |
| Eritrea | Afr | VAM | 1332 (719, 2323) | 18 (10, 32) |
| Ethiopia | Afr | VAM | 56137 (26676, 109535) | 28 (13, 55) |
| Fiji | Wpr | VRM | 26 (15, 42) | 16 (9, 26) |
| Gabon | Afr | VAM | 285 (172, 471) | 15 (9, 24) |
| Gambia | Afr | VAM | 667 (361, 1159) | 16 (9, 28) |
| Georgia | Eur | VAM | 162 (101, 260) | 26 (16, 42) |
| Ghana | Afr | VAM | 5125 (2611, 9412) | 16 (8, 29) |
| Grenada | Amr | VRM | 1 (0, 1) | 18 (11, 28) |
| Guatemala | Amr | VAM | 1330 (768, 2206) | 12 (7, 21) |
| Guinea | Afr | VAM | 6782 (4735, 9714) | 18 (13, 26) |
| Guinea-Bissau | Afr | VAM | 1706 (1207, 2411) | 18 (13, 26) |
| Guyana | Amr | VAM | 86 (59, 123) | 17 (12, 24) |
| Haiti | Amr | VAM | 2872 (1548, 5328) | 23 (12, 43) |
| Honduras | Amr | VAM | 397 (168, 836) | 13 (6, 28) |
| India | Sear | VAM | 264766 (188765, 371365) | 32 (23, 45) |
| Indonesia | Sear | VAM | 21281 (13579, 33354) | 23 (15, 36) |
| Iran (Islamic Republic of) | Emr | VRM | 4259 (2379, 7625) | 22 (12, 39) |
| Iraq | Emr | VAM | 4573 (3145, 6650) | 27 (18, 39) |
| Jamaica | Amr | VAM | 119 (60, 221) | 10 (5, 18) |
| Jordan | Emr | VRM | 240 (103, 499) | 22 (10, 46) |
| Kazakhstan | Eur | VRM | 440 (199, 924) | 10 (4, 20) |
| Kenya | Afr | VAM | 30494 (14501, 59271) | 22 (10, 43) |
| Kiribati | Wpr | VAM | 10 (6, 15) | 15 (10, 23) |
| Kyrgyzstan | Eur | VAM | 501 (333, 755) | 20 (13, 30) |
| Lao People's Dem. Republic | Wpr | VAM | 1770 (857, 3655) | 26 (13, 54) |
| Lebanon | Emr | VRM | 67 (30, 136) | 19 (8, 38) |
| Lesotho* | Afr | VAM | 904 (456, 1584) | 38 (19, 67) |
| Liberia | Afr | VAM | 2138 (1383, 3238) | 15 (10, 23) |
| Libyan Arab Jamahiriya | Emr | VRM | 157 (77, 296) | 15 (7, 28) |
| Madagascar | Afr | VAM | 11817 (7570, 18359) | 25 (16, 39) |
| Malawi* | Afr | VAM | 4779 (3168, 7210) | 12 (8, 18) |
| Malaysia | Wpr | VRM | 200 (103, 362) | 11 (6, 20) |
| Maldives | Sear | VAM | 16 (10, 26) | 24 (15, 39) |
| Mali | Afr | VAM | 12802 (8585, 18735) | 18 (12, 26) |
| Marshall Islands | Wpr | VAM | 4 (2, 7) | 15 (9, 24) |
| Mauritania | Afr | VAM | 2437 (1609, 3690) | 32 (21, 49) |
| Micronesia (Fed. States of) | Wpr | VAM | 10 (6, 16) | 15 (9, 24) |
| Monaco | Eur | VRM | 0 (0, 0) | 0 (0, 0) |
| Mongolia | Wpr | VAM | 185 (113, 292) | 14 (8, 22) |
| Montenegro | Eur | VRM | 2 (1, 3) | 8 (6, 12) |
| Morocco | Emr | VAM | 2040 (1187, 3339) | 24 (14, 39) |
| Mozambique* | Afr | VAM | 11132 (7468, 16592) | 15 (10, 23) |
| Myanmar | Sear | VAM | 18132 (9956, 33025) | 37 (20, 68) |
| Namibia* | Afr | VAM | 257 (146, 456) | 18 (10, 33) |
| Nauru | Wpr | VAM | 1 (1, 2) | 38 (26, 55) |
| Nepal | Sear | VAM | 4559 (3250, 6394) | 32 (23, 45) |
| Nicaragua | Amr | VAM | 337 (164, 637) | 16 (8, 31) |
| Niger | Afr | VAM | 17714 (12693, 24722) | 19 (13, 26) |
| Nigeria | Afr | VAM | 148550 (105218, 209727) | 19 (14, 27) |
| Oman | Emr | VRM | 34 (16, 65) | 13 (6, 24) |
| Pakistan | Emr | VAM | 70620 (55171, 90397) | 39 (31, 50) |
| Palau | Wpr | VRM | 0 (0, 0) | 0 (0, 0) |
| Papua New Guinea | Wpr | VAM | 2045 (1263, 3310) | 24 (15, 38) |
| Paraguay | Amr | VAM | 363 (201, 627) | 18 (10, 32) |
| Peru | Amr | VRM | 976 (575, 1581) | 14 (8, 22) |
| Philippines | Wpr | VAM | 8355 (4997, 13971) | 21 (13, 35) |
| Qatar | Emr | VRM | 3 (3, 3) | 4 (4, 4) |
| Rwanda | Afr | VAM | 7249 (3502, 13785) | 27 (13, 51) |
| Samoa | Wpr | VAM | 10 (6, 16) | 16 (9, 27) |
| Sao Tome and Principe | Afr | VAM | 89 (59, 135) | 27 (18, 41) |
| Saudi Arabia | Emr | VRM | 586 (290, 1095) | 11 (5, 20) |
| Senegal | Afr | VAM | 5456 (3042, 9301) | 17 (9, 28) |
| Sierra Leone | Afr | VAM | 5183 (3459, 7635) | 16 (11, 23) |
| Solomon Islands | Wpr | VAM | 76 (30, 179) | 23 (9, 55) |
| Somalia | Emr | VAM | 16856 (10535, 26969) | 32 (20, 52) |
| South Africa | Afr | VRM | 10553 (3909, 16641) | 21 (8, 32) |
| Sri Lanka | Sear | VRM | 873 (427, 1786) | 36 (18, 74) |
| Sudan | Emr | VAM | 26467 (14109, 46881) | 31 (16, 55) |
| Suriname | Amr | VAM | 21 (11, 37) | 15 (8, 26) |
| Swaziland* | Afr | VAM | 144 (80, 259) | 6 (4, 12) |
| Syrian Arab Republic | Emr | VRM | 1192 (490, 2599) | 24 (10, 53) |
| Tajikistan | Eur | VAM | 1397 (1004, 1927) | 18 (13, 25) |
| Thailand | Sear | VRM | 1014 (512, 2010) | 25 (13, 49) |
| Timor-Leste | Sear | VAM | 776 (413, 1455) | 38 (20, 72) |
| Togo | Afr | VAM | 1991 (1246, 3154) | 15 (10, 24) |
| Tonga | Wpr | VRM | 5 (3, 8) | 16 (10, 26) |
| Tunisia | Emr | VRM | 384 (210, 702) | 25 (14, 46) |
| Turkey | Eur | VRM | 796 (370, 1559) | 8 (4, 15) |
| Turkmenistan | Eur | VAM | 617 (432, 881) | 20 (14, 29) |
| Uganda | Afr | VAM | 20290 (11923, 33403) | 14 (8, 23) |
| United Arab Emirates | Emr | VRM | 7 (7, 7) | 4 (4, 4) |
| United Republic of Tanzania | Afr | VAM | 20015 (13280, 30165) | 17 (11, 26) |
| Uzbekistan | Eur | VAM | 2280 (1518, 3424) | 22 (15, 33) |
| Vanuatu | Wpr | VAM | 34 (14, 85) | 25 (10, 63) |
| Viet Nam | Wpr | VRM | 1619 (1011, 2595) | 21 (13, 33) |
| Yemen | Emr | VAM | 8317 (4237, 15469) | 28 (14, 52) |
| Zambia* | Afr | VAM | 7817 (5553, 11003) | 14 (10, 19) |
| Zimbabwe* | Afr | VAM | 3093 (2060, 4645) | 12 (8, 19) |
