# Supplementary material for: Estimating Pneumonia Deaths of Post-Neonatal Children in Countries of Low or No Death Certification in 2008
Source: PLoS One. 2011 Sep 22;6(9):e25095. doi: 10.1371/journal.pone.0025095 (PMC3178589; doi:10.1371/journal.pone.0025095)
Supplement: Table S2 — Differences in the national and regional pneumonia mortality single and multi-cause estimates (Emr: Eastern Mediterranean Region; Eur: Europe Region, Afr: Africa Region; Amr: Americas Region; Sear: South East Asia Region; Wpr: Western Pacific Region; PN: Pneumonia). (DOC) [file pone.0025095.s003.doc]

Supplementary table S2 Differences in the national and regional pneumonia mortality single and multi-cause estimates (Emr: Eastern Mediterranean Region; Eur: Europe Region, Afr: Africa Region; Amr: Americas Region; Sear: South East Asia Region; Wpr: Western Pacific Region; PN: Pneumonia)

| **Country** | **WHO region** | **1-59 mo deaths** | **Single-cause model** | | **Multi-cause model** | |
| --- | --- | --- | --- | --- | --- | --- |
|  |  |  | **% of 1-59m PN deaths over 1-59m total deaths** | **# of 1-59m PN deaths** | **% of 1-59m PN deaths over 1-59m total deaths** | **# of 1-59m PN deaths** |
| Afghanistan | Emr | 247615 | 26.61 | 65898 | 29.37 | 72716 |
| Albania | Eur | 485 | 11.54 | 56 | 21.86 | 106 |
| Algeria | Afr | 12925 | 21.00 | 2715 | 28.65 | 3703 |
| Andorra | Eur | 2 | 0.00 | 0 | 3.94 | 0 |
| Angola | Afr | 129045 | 20.46 | 26409 | 23.18 | 29910 |
| Antigua and Barbuda | Amr | 6 | 8.71 | 1 | 12.08 | 1 |
| Armenia | Eur | 465 | 12.05 | 56 | 26.12 | 121 |
| Azerbaijan | Eur | 3060 | 21.31 | 652 | 35.05 | 1072 |
| Bangladesh | Sear | 69053 | 32.51 | 22448 | 28.82 | 19902 |
| Benin | Afr | 28117 | 16.22 | 4562 | 19.94 | 5607 |
| Bhutan | Sear | 676 | 38.78 | 262 | 33.57 | 227 |
| Bolivia | Amr | 7663 | 18.77 | 1438 | 29.59 | 2268 |
| Bosnia and Herzegovina | Eur | 227 | 9.50 | 22 | 21.65 | 49 |
| Botswana | Afr | 706 | 19.92 | 141 | 14.50 | 102 |
| Burkina Faso | Afr | 91374 | 15.26 | 13944 | 21.27 | 19432 |
| Burundi | Afr | 33124 | 20.96 | 6943 | 20.27 | 6714 |
| Cambodia | Wpr | 21089 | 23.73 | 5005 | 40.48 | 8537 |
| Cameroon | Afr | 67523 | 16.55 | 11172 | 21.26 | 14358 |
| Cape Verde | Afr | 149 | 48.76 | 73 | 21.30 | 32 |
| Central African Republic | Afr | 18682 | 17.14 | 3202 | 22.93 | 4284 |
| Chad | Afr | 76540 | 17.44 | 13351 | 21.40 | 16377 |
| China | Wpr | 159709 | 20.17 | 32213 | 34.45 | 55026 |
| Comoros | Afr | 1377 | 27.71 | 382 | 31.06 | 428 |
| Congo | Afr | 11234 | 17.32 | 1945 | 19.36 | 2175 |
| Cote d'Ivoire | Afr | 49899 | 16.97 | 8469 | 18.10 | 9031 |
| Cyprus | Eur | 21 | 10.77 | 2 | 4.31 | 1 |
| Democratic People's Republic of Korea | Sear | 8873 | 24.83 | 2203 | 34.00 | 3017 |
| Democratic Republic of the Congo | Afr | 390839 | 20.27 | 79222 | 22.55 | 88119 |
| Djibouti | Emr | 1380 | 36.17 | 499 | 25.13 | 347 |
| Dominican Republic | Amr | 3190 | 14.35 | 458 | 28.71 | 916 |
| Ecuador | Amr | 3832 | 14.52 | 556 | 22.21 | 851 |
| Egypt | Emr | 17631 | 27.59 | 4864 | 16.78 | 2958 |
| El Salvador | Amr | 1319 | 14.64 | 193 | 17.40 | 230 |
| Equatorial Guinea | Afr | 2482 | 19.08 | 474 | 14.20 | 352 |
| Eritrea | Afr | 7205 | 18.49 | 1332 | 24.01 | 1730 |
| Ethiopia | Afr | 199839 | 28.09 | 56137 | 19.76 | 39493 |
| Fiji | Wpr | 164 | 15.86 | 26 | 16.99 | 28 |
| Gabon | Afr | 1930 | 14.74 | 285 | 12.44 | 240 |
| Gambia | Afr | 4172 | 15.99 | 667 | 16.95 | 707 |
| Georgia | Eur | 620 | 26.15 | 162 | 34.43 | 213 |
| Ghana | Afr | 32052 | 15.99 | 5125 | 12.82 | 4109 |
| Grenada | Amr | 3 | 18.03 | 1 | 17.46 | 1 |
| Guatemala | Amr | 10647 | 12.50 | 1330 | 25.16 | 2679 |
| Guinea | Afr | 37288 | 18.19 | 6782 | 17.91 | 6677 |
| Guinea-Bissau | Afr | 9254 | 18.43 | 1706 | 21.27 | 1968 |
| Guyana | Amr | 511 | 16.84 | 86 | 23.74 | 121 |
| Haiti | Amr | 12432 | 23.10 | 2872 | 28.20 | 3505 |
| Honduras | Amr | 3030 | 13.10 | 397 | 27.32 | 828 |
| India | Sear | 826060 | 32.05 | 264752 | 24.65 | 203588 |
| Indonesia | Sear | 92895 | 22.91 | 21281 | 33.01 | 30665 |
| Iran (Islamic Republic of) | Emr | 19424 | 21.93 | 4259 | 26.41 | 5130 |
| Iraq | Emr | 17065 | 26.80 | 4573 | 30.72 | 5242 |
| Jamaica | Amr | 1197 | 9.93 | 119 | 27.49 | 329 |
| Jordan | Emr | 1077 | 22.25 | 240 | 17.16 | 185 |
| Kazakhstan | Eur | 4595 | 9.58 | 440 | 28.29 | 1300 |
| Kenya | Afr | 138896 | 21.95 | 30494 | 20.28 | 28166 |
| Kiribati | Wpr | 65 | 15.11 | 10 | 31.21 | 20 |
| Kyrgyzstan | Eur | 2538 | 19.74 | 501 | 33.18 | 842 |
| Lao People's Democratic Republic | Wpr | 6768 | 26.16 | 1770 | 38.95 | 2636 |
| Lebanon | Emr | 361 | 18.51 | 67 | 10.52 | 38 |
| Lesotho | Afr | 2358 | 38.34 | 904 | 21.54 | 508 |
| Liberia | Afr | 13904 | 15.38 | 2138 | 20.48 | 2848 |
| Libyan Arab Jamahiriya | Emr | 1071 | 14.69 | 157 | 12.28 | 132 |
| Madagascar | Afr | 47122 | 25.08 | 11817 | 28.25 | 13311 |
| Malawi | Afr | 39155 | 12.21 | 4779 | 15.95 | 6243 |
| Malaysia | Wpr | 1771 | 11.29 | 200 | 5.55 | 98 |
| Maldives | Sear | 66 | 23.90 | 16 | 24.34 | 16 |
| Mali | Afr | 71940 | 17.80 | 12802 | 18.95 | 13632 |
| Marshall Islands | Wpr | 28 | 15.00 | 4 | 30.19 | 8 |
| Mauritania | Afr | 7514 | 32.43 | 2437 | 23.84 | 1791 |
| Micronesia (Federated States of) | Wpr | 67 | 15.12 | 10 | 41.87 | 28 |
| Monaco | Eur | 1 | 0.00 | 0 | 3.25 | 0 |
| Mongolia | Wpr | 1352 | 13.69 | 185 | 36.39 | 492 |
| Montenegro | Eur | 24 | 8.30 | 2 | 13.08 | 3 |
| Morocco | Emr | 8505 | 23.98 | 2040 | 25.95 | 2207 |
| Mozambique | Afr | 72855 | 15.28 | 11132 | 19.32 | 14073 |
| Myanmar | Sear | 48910 | 37.07 | 18132 | 19.22 | 9403 |
| Namibia | Afr | 1391 | 18.48 | 257 | 16.55 | 230 |
| Nauru | Wpr | 3 | 37.71 | 1 | 40.05 | 1 |
| Nepal | Sear | 14244 | 32.00 | 4559 | 26.87 | 3827 |
| Nicaragua | Amr | 2084 | 16.18 | 337 | 27.44 | 572 |
| Niger | Afr | 94064 | 18.83 | 17714 | 23.93 | 22512 |
| Nigeria | Afr | 768080 | 19.34 | 148550 | 20.73 | 159222 |
| Oman | Emr | 268 | 12.58 | 34 | 8.77 | 24 |
| Pakistan | Emr | 180736 | 39.07 | 70620 | 28.89 | 52221 |
| Palau | Wpr | 3 | 0.00 | 0 | 12.00 | 0 |
| Papua New Guinea | Wpr | 8665 | 23.60 | 2045 | 32.11 | 2782 |
| Paraguay | Amr | 1979 | 18.36 | 363 | 26.79 | 530 |
| Peru | Amr | 7121 | 13.71 | 976 | 20.67 | 1472 |
| Philippines | Wpr | 39972 | 20.90 | 8355 | 33.88 | 13544 |
| Qatar | Emr | 88 | 3.78 | 3 | 5.86 | 5 |
| Rwanda | Afr | 27037 | 26.81 | 7249 | 20.60 | 5569 |
| Samoa | Wpr | 61 | 15.86 | 10 | 25.67 | 16 |
| Sao Tome and Principe | Afr | 326 | 27.41 | 89 | 35.08 | 114 |
| Saudi Arabia | Emr | 5527 | 10.61 | 586 | 13.45 | 743 |
| Senegal | Afr | 32898 | 16.58 | 5456 | 19.16 | 6302 |
| Sierra Leone | Afr | 32712 | 15.84 | 5183 | 21.34 | 6979 |
| Solomon Islands | Wpr | 327 | 23.16 | 76 | 34.83 | 114 |
| Somalia | Emr | 51955 | 32.44 | 16856 | 25.11 | 13048 |
| South Africa | Afr | 51237 | 20.60 | 10553 | 8.05 | 4126 |
| Sri Lanka | Sear | 2424 | 36.00 | 873 | 12.02 | 291 |
| Sudan | Emr | 85825 | 30.84 | 26467 | 15.29 | 13120 |
| Suriname | Amr | 143 | 14.71 | 21 | 16.64 | 24 |
| Swaziland | Afr | 2235 | 6.44 | 144 | 11.85 | 265 |
| Syrian Arab Republic | Emr | 4932 | 24.16 | 1192 | 15.89 | 784 |
| Tajikistan | Eur | 7714 | 18.11 | 1397 | 30.28 | 2336 |
| Thailand | Sear | 4064 | 24.95 | 1014 | 11.75 | 478 |
| Timor-Leste | Sear | 2023 | 38.34 | 776 | 18.90 | 382 |
| Togo | Afr | 12965 | 15.36 | 1991 | 14.86 | 1927 |
| Tonga | Wpr | 31 | 16.06 | 5 | 18.51 | 6 |
| Tunisia | Emr | 1527 | 25.16 | 384 | 15.40 | 235 |
| Turkey | Eur | 10551 | 7.54 | 796 | 23.66 | 2497 |
| Turkmenistan | Eur | 3014 | 20.46 | 617 | 36.23 | 1092 |
| Uganda | Afr | 144983 | 13.99 | 20290 | 16.24 | 23541 |
| United Arab Emirates | Emr | 166 | 4.02 | 7 | 4.80 | 8 |
| United Republic of Tanzania | Afr | 115717 | 17.30 | 20015 | 19.03 | 22019 |
| Uzbekistan | Eur | 10308 | 22.12 | 2280 | 33.89 | 3493 |
| Vanuatu | Wpr | 134 | 25.32 | 34 | 32.66 | 44 |
| Viet Nam | Wpr | 7854 | 20.62 | 1619 | 12.48 | 980 |
| Yemen | Emr | 29554 | 28.14 | 8317 | 25.00 | 7390 |
| Zambia | Afr | 57255 | 13.65 | 7817 | 17.81 | 10198 |
| Zimbabwe | Afr | 24825 | 12.46 | 3093 | 17.26 | 4286 |
| All 122 countries |  | 5056065 | 23.36 | 1181037 | 23.01 | 1163552 |
| Countries from Africa WHO region (n=44 of 46) |  | 2965225 | 19.22 | 569940 | 20.42 | 605563 |
| Countries from America WHO region (n=15 of 35) |  | 55157 | 16.59 | 9149 | 25.97 | 14326 |
| Countries from East Mediterranean WHO region (n=19 of 21) |  | 674707 | 30.69 | 207061 | 26.23 | 176964 |
| Countries from Europe WHO region (n=15 of 53) |  | 43625 | 16.01 | 6983 | 30.09 | 13127 |
| Countries from South East Asia WHO region (n=11 of 11) |  | 1069288 | 31.45 | 336329 | 25.88 | 276782 |
| Countries from West Pacific WHO region (n=18 of 27) |  | 248063 | 20.79 | 51575 | 34.55 | 85694 |
