# Supplementary figures and images for: Estimating Pneumonia Deaths of Post-Neonatal Children in Countries of Low or No Death Certification in 2008
Source: PLoS One. 2011 Sep 22;6(9):e25095. doi: 10.1371/journal.pone.0025095 (PMC3178589; doi:10.1371/journal.pone.0025095)

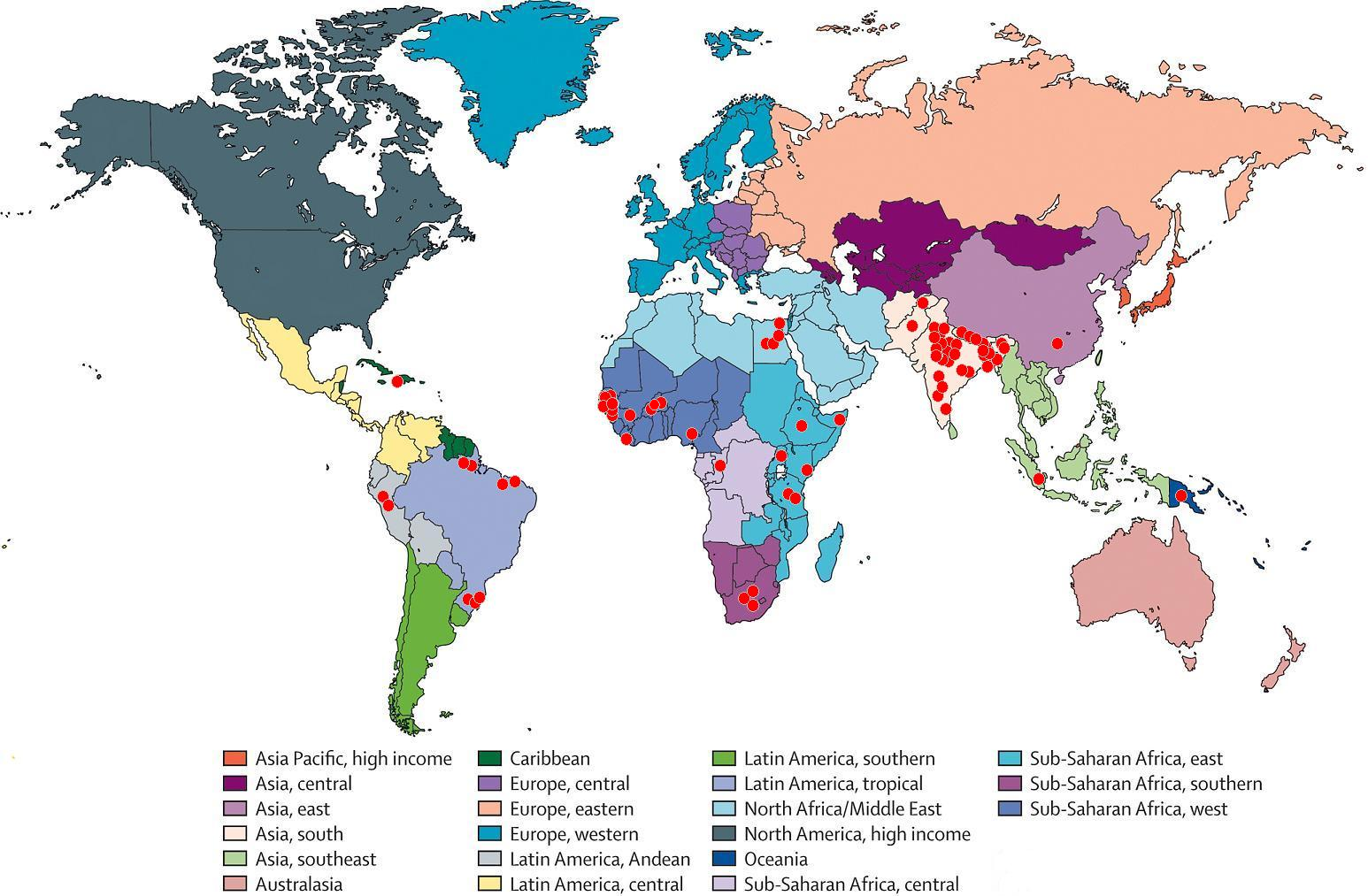

Supplement: Figure S1 — Distribution of the 81 data points (58 verbal autopsy studies) that were used for the development of the single cause models. (TIF) [file pone.0025095.s004.tif]

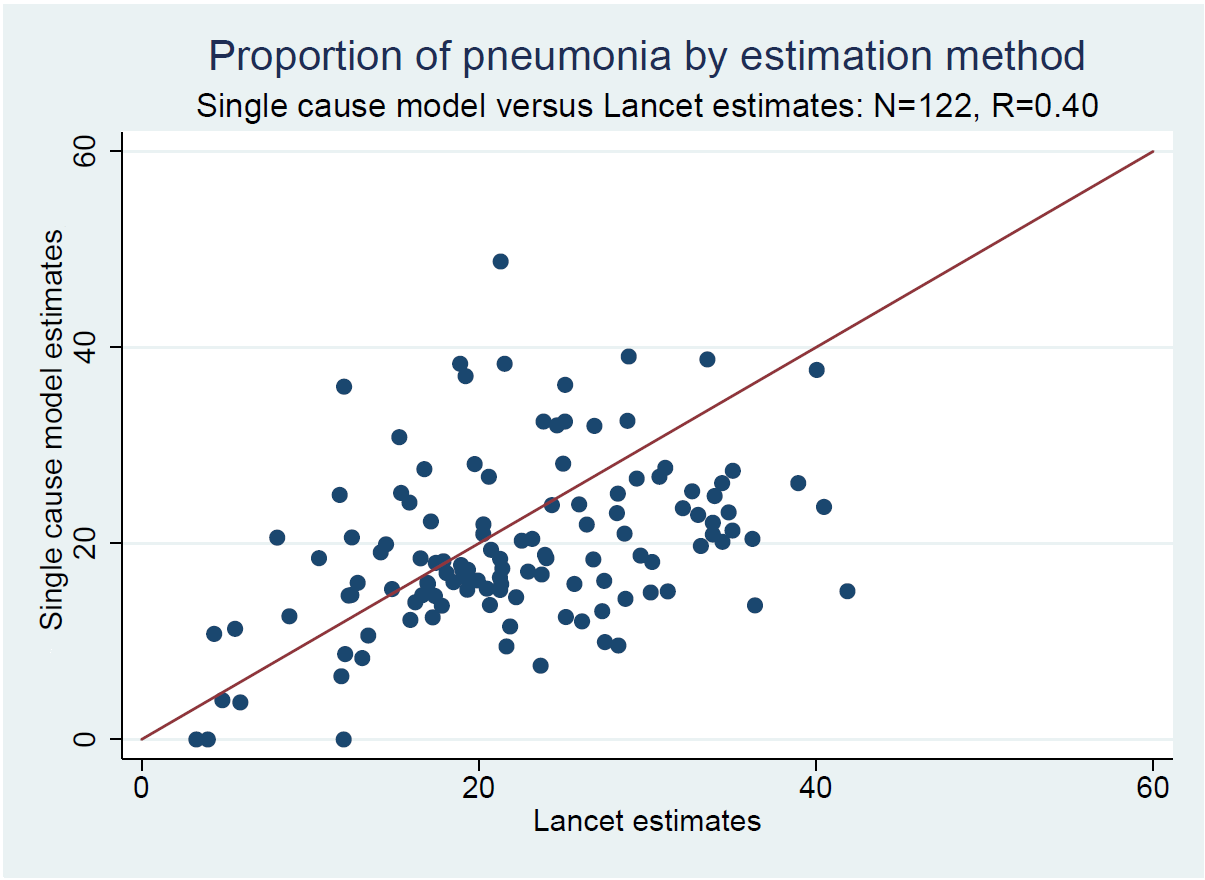

Supplement: Figure S2 — Comparison of post-neonatal pneumonia estimates for 122 countries between the single-cause and multi-cause model estimates (as published in Black et al, 2010). (TIF) [file pone.0025095.s005.tif]
